# AI-augmented versus expert-authored multiple-choice questions: a psychometric comparison in a high-stakes specialty examination

**DOI:** 10.1186/s12909-026-10082-4

**Published:** 2026-08-07

**Authors:** Wilma Anschuetz, Daniel Stricker, Claudia Canonica, Céline Montavon, Christian De Geyter, Sören Huwendiek

**Affiliations:** 1 Department for Assessment and Evaluation, Institute for Medical Education (IML), University of Bern, Bern, Switzerland; 2 Master of Medical Education Program (MME), Institute for Medical Education (IML), University of Bern, Bern, Switzerland; 3 Department of Obstetrics and Gynecology, Regional Hospital of Bellinzona, EOC (Ente Ospedaliero Cantonale), Lugano, Switzerland; 4 Department of Gynecology and Gynecological Oncology, University Hospital Basel, University of Basel, Basel, Switzerland; 5 Reproductive Medicine and Gynecological Endocrinology (RME), University Hospital Basel, University of Basel, Basel, Switzerland

**Keywords:** Artificial intelligence, Medical education, High-stakes assessment, Multiple-choice questions, Psychometrics, Distractor functioning, Specialty examination

## Abstract

**Background:**

Multiple-choice questions (MCQs) are widely used in written assessments, particularly in high-stakes medical examinations. Developing high-quality MCQs is time-consuming and requires subject matter expertise. Large language models (LLMs), such as ChatGPT-4o, have therefore been proposed as tools to support item generation. Prior studies have examined AI-generated MCQs in formative or simulated contexts; evidence from authentic high-stakes examinations remains limited. This study compared the psychometric performance of AI-augmented and expert-authored MCQs within a national specialty examination.

**Methods:**

For a national high-stakes examination, 90 AI-augmented items (generated with ChatGPT-4o) and 25 expert-authored items were developed and underwent expert screening, revision, and blinded review board evaluation. The examination was administered to 304 candidates (166 basic-level, 138 specialist-level). Item analysis included 12 AI-augmented items, 14 expert-authored items, and 89 previously used bank items. Psychometric performance was assessed using classical test theory (item difficulty, discrimination, internal consistency, distractor functioning). Differences across item sources and candidate groups were analyzed using ANOVA with post-hoc testing.

**Results:**

AI-augmented items were easier than both newly expert-authored items and previously used bank items, as reflected by a significantly higher proportion of correct responses (mean P-value: 84,7% vs. 73,5% and 69,4%, respectively; (F_(2,604)_= 297.187, p<0.001)). Discrimination indices were comparable across item sources. Distractor analysis showed a higher proportion of AI-augmented items with no functioning distractor (*p* < 0.05). Overall test reliability did not significantly change after inclusion of AI-augmented items.

**Conclusions:**

In this study, AI-augmented MCQs could be integrated into a high-stakes examination following rigorous multi-stage review, without evidence of detrimental effects on overall test performance. However, even after expert revision, such items tended to be easier and showed weaker distractor functioning. The substantial exclusion rate and systematic differences in item difficulty highlight the need for structured quality assurance and difficulty calibration when integrating AI-augmented item development into specialty-level assessments.

## Background

High-stakes examinations in medical education commonly rely on multiple-choice questions (MCQs) because they enable standardized, scalable, and objective assessment of knowledge and understanding across large cohorts [1]. Higher certification examination scores have been shown to be associated with improved patient outcomes [2]. As part of quality assurance and to reflect current scientific and disciplinary developments, examinations require the regular introduction of new items. This necessitates the development of numerous new questions each year. The development of valuable MCQs with authentic clinical case vignettes and appropriate distractors (incorrect answer options) requires the consideration of numerous aspects and expertise [3, 4]. Recruiting clinical experts willing to engage in item development remains challenging, particularly in the context of high clinical workload [5].

Recently, generative large language models (LLMs) such as ChatGPT have been proposed as potential tools to accelerate item development. Early studies indicate that while artificial intelligence (AI) can substantially reduce drafting time for MCQs, it may also introduce risks related to content accuracy and inappropriate cognitive complexity [6–9]. Studies in formative contexts reported acceptable psychometric properties of AI-generated type A MCQs, including moderate difficulty and good discrimination [10], findings that were later extended to clinically complex items in a blinded mock examination, where GPT-4-generated questions showed comparable psychometric performance to expert-authored items but lower distractor efficiency [11]. A review of ChatGPT prompt strategies in medical education highlighted that prompt specificity, role- or persona-framing, and iterative prompt refinement significantly influence the accuracy and educational usefulness of AI-generated items, while also warning that insufficiently controlled prompts may result in hallucinations, superficial content, or clinical inaccuracies [12, 13].

Comparative studies of AI-generated and expert-authored MCQs in formative assessments at the postgraduate level have shown that AI-generated items tend to be easier and primarily assess lower-order cognitive skills. While discrimination indices were largely comparable across item sources, AI-generated questions demonstrated a higher incidence of factual inaccuracies and psychometric concerns, reinforcing that human oversight remains essential [6, 14]. Across studies, expert review has been emphasized to ensure factual accuracy, cognitive complexity, and alignment with examination blueprints before AI-generated MCQs can be used in high-stakes settings [6, 7, 12, 15].

While formative and mock-examination studies suggest acceptable psychometric properties of AI-generated MCQs, evidence from authentic high-stakes examinations with large candidate cohorts remains scarce. In particular, little is known about how AI-augmented items perform after structured expert review when directly compared with newly authored expert items and previously used bank items within the same examination. Moreover, previous research has rarely examined whether performance characteristics of AI-augmented items differ across candidate groups at different stages of postgraduate training. This is particularly interesting given that optimal item discrimination depends on aligning item difficulty with candidates’ level of expertise, raising the question of whether AI-augmented items are differentially suited to specific groups at different stages of postgraduate training [16].

Considering these developments, this study addressed the following research questions: (1) whether new AI-augmented items differ from newly expert-authored and previously used bank items in core psychometric characteristics, specifically item difficulty and discrimination; (2) how AI-augmented items compare with newly expert-authored and previously used bank items in terms of functioning distractors; and (3) how these performance characteristics vary across candidate groups at different stages of training (basic versus specialist candidates).

By providing data from an authentic item generation process and examination context, this study contributes to future governance decisions regarding the safe and responsible integration of AI into MCQ assessment practice.

## Methods

### Candidate groups and examination characteristics

The examination in gynecology and obstetrics (OB/GYN) was administered to two predefined candidate groups: basic examination candidates (*n* = 166) and specialist examination candidates (*n* = 138), resulting in a total of 304 candidates. Overall, 244 (80.3%) candidates were female (basic group: 81.9%; specialist group: 78.3%). Age and time since graduation were not normally distributed, median age was 30.3 years (basic group: 29.7 years; specialist group: 31.2 years), and median time since graduation was 3.7 years (basic group: 2.7 years; specialist group: 4.6 years). For the national specialist examination, candidates are required to take the written examinations at two different stages of their postgraduate training. The first stage, referred to as the basic examination, is typically taken after approximately two years of clinical experience as a junior resident. The final specialist examination is generally completed after around three to four years of training. These timelines represent recommended stages rather than mandatory requirements, and actual timing may vary. Basic examination candidates are therefore expected to demonstrate foundational subject-specific knowledge, whereas specialist examination candidates are expected at a more advanced stage. This staged examination pathway allowed for differential analysis of item metrics by candidate seniority, providing insight into how AI-augmented and expert-authored items performed across varying levels of subject-specific expertise. The examination consisted of 120 MCQs: 81 single-best answer questions (A-type) and 39 multiple-true-false questions (K-prime). Single-best answer questions had 4 or 5 answer options. Both candidate groups completed the same set of questions under standard examination conditions.

### New AI-augmented items

For the generation of new AI-augmented items, OpenAI’s ChatGPT was accessed via the browser-based web interface using the GPT-4o model. A dedicated ChatGPT Teams account was created and used exclusively for this study, with memory functions disabled. This approach was chosen to evaluate model outputs under conditions that reflect typical use by question authors without technical expertise or programming skills. All prompts were entered manually using default system settings. No external tools, plugins, or file uploads were enabled.

Prior to item generation, the prompt was developed through an iterative refinement process to define the expected level of clinical complexity, vignette length, and structure of answer options. The development process was informed by published recommendations on AI-supported multiple-choice question construction [6, 7, 12]. The goal was to establish a generic and reusable prompt template capable of consistently producing structurally sound MCQ items that test advanced cognitive skills. The template was designed to be adaptable across different learning objectives and topic areas, while minimizing the need for subsequent human modification.

Prompt revisions were undertaken in a stepwise manner. After each iteration, the generated items were reviewed qualitatively, and adjustments to the prompt were made where necessary. Following each prompt modification, a new chat session was initiated to avoid potential influences of prior conversational context on subsequent outputs. This process continued until additional refinements no longer resulted in systematic improvements in item quality.

The final prompt template included the following elements:

- Contextual framing (examination setting, target learner population).
- Requirements for clinical vignette and question stem.
- Answer structure specifications (number and homogeneity of answer options, and plausibility of distractors).
- Explanatory feedback (with reasoning for correct answer and distractors).
- Evidence integration (reference literature).
- Exemplary item and structural requirements.

As the content of the examination is not publicly disclosed and considering unresolved data security concerns associated with the use of GPT-4o, no detailed information regarding the examination blueprint was provided to GPT-4o. Initially, the subject area was introduced in broad terms using “gynecology and obstetrics” or “reproductive medicine and gynecologic endocrinology”. The initial output, consisting of 45 questions, already covered a wide range of different topics, although not all desired aspects of content were represented. Consequently, following prompts targeted specific subject areas in order to ensure the broadest possible thematic diversity of the generated questions. However, it became evident that GPT-4o was unable to generate questions in certain domains, such as sexual medicine. Further, the variety and number of adequate questions were limited in important topics as emergency situations, gynecological infectious diseases or preeclampsia. During an initial screening, items not meeting the established quality criteria for the OB/GYN examination were excluded [3, 17]. Exclusion took place because of low cognitive complexity (*n* = 27); structural flaws, including cueing and trivial or non-plausible distractors (*n* = 24); factual inaccuracies, clinically irrelevant content or unclear clinical vignettes (*n* = 12); and duplication of existing content (*n* = 7). Items could be excluded for more than one reason. Items that met the predefined quality criteria were subjected to a formal pre-revision process, primarily to eliminate irrelevant or potentially misleading information and to identify potential issues ahead of the subsequent expert review. In total, 90 draft items had to be produced to obtain 45 AI-generated items suitable for submission to the review board.

### New expert-authored items

New expert-authored items were developed during a structured item-writing workshop. Eight OB/GYN experts individually prepared draft questions aligned with predefined learning objectives and were provided with guidance on best practices in MCQ construction, including vignette structure, distractor plausibility, and avoidance of common item-writing flaws. Draft items were screened and formally pre-revised by an assessment expert, drawing on established principles of MCQ design [3, 17]. During the workshop, items were revised in small groups (two to three OB/GYN experts plus one assessment expert) through collaborative consensus based on the revision suggestions. During the workshop, a total of 25 new questions were developed, of which 23 were accepted for further processing.

### Review process

**Fig. 1:**
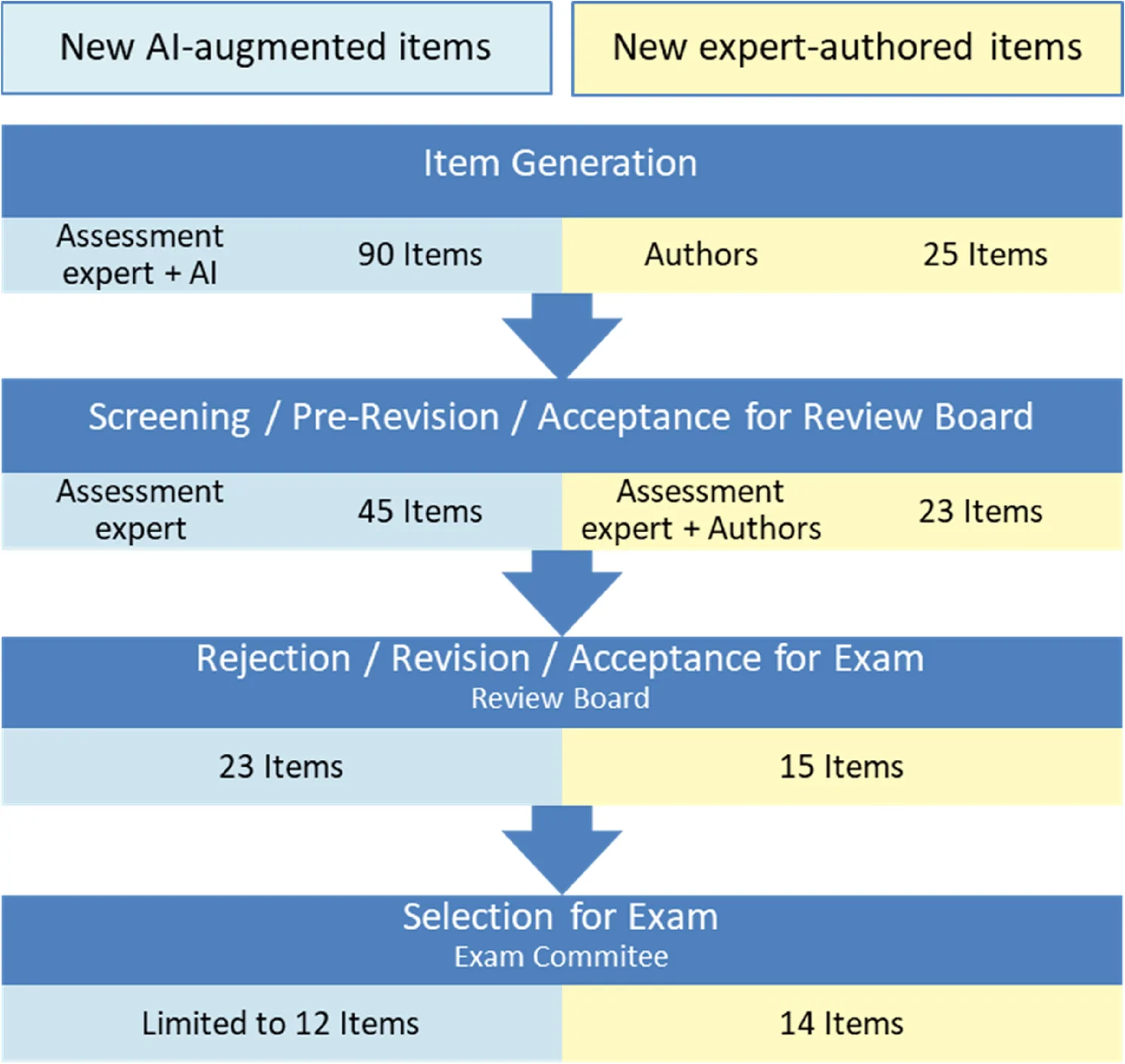
Flow diagram of item generation and selection for AI-augmented versus expert-authored items. Numbers indicate items retained at each stage of screening, review, and final exam selection

Figure 1 provides an overview of the development, screening, and selection process for both AI-augmented and expert-authored items. In total, 68 new items (45 AI-generated and 23 expert-authored) meeting formal criteria were selected for evaluation by the national OB/GYN review board. The review board consists of approximately 15 experts, five for each of the 3 subspecialties (1. Gynecologic endocrinology and reproductive medicine; 2. General and surgical gynecology; 3. Obstetrics). Experts from the review board were informed about the introduction of AI-augmented items but blinded to the origin of each item. For each item, they assessed clinical plausibility and accuracy, cognitive complexity, and overall suitability for high-stakes examination use. Review discussions were moderated by an assessment expert to ensure a structured and standardized decision-making process. In cases of substantial disagreement, for example due to differing therapeutic approaches across institutions, items were discussed until consensus on an appropriate revision was achieved. If substantial concerns could not be resolved through revision, the item was excluded.

### Integration into the examination

Although 23 AI-augmented items were approved by the review board, the exam committee limited the number of AI-augmented items in the examination to a maximum of twelve in the first implementation. Twelve AI-augmented items were therefore selected to achieve an even distribution across the blueprint. Among eligible items, preference was given to those judged to be of the highest educational and clinical quality. One of 15 approved expert-authored items had to be excluded from examination composition due to duplicate content. Both AI-assisted and expert-authored items were embedded throughout the examination booklet and presented in identical format. No indication of item origin was provided, ensuring that candidates remained blinded to AI involvement.

### Previously used expert-authored bank items

Previously used items were drawn from an established item bank maintained by the Swiss national OB/GYN-specialist association Gynécologie Suisse. These 94 items had previously been authored by experts, either individually or in item-writing workshops and had undergone review and approval by the review board, post-examination statistical analysis, and evaluation of candidate feedback. Items flagged for statistical or content concerns in prior examinations had been revised before re-approval. The selection of items followed the predefined blueprint specifications and was guided by the aim of achieving a good distribution of item difficulties aligned with the expected ability level of the candidates. Five bank items had to be eliminated during post-exam analysis due to factual or formal reasons and were not included in this study, resulting in a total of 89 items.

### Psychometric analysis

All statistical analyses were performed using SPSS for Windows, version 30 (IBM, Armonk, NY, USA), with significance set at *p* < 0.05. Effect size is reported by means of partial eta-squared, where values below 0.07 denote a small effect, from 0.07 to 0.14 a moderate and above 0.14 a large effect.

Psychometric analysis was based on classical test theory [18]. Item difficulty (P-value, indicating the proportion of correct answers) and discrimination index (corrected item-total correlation) were calculated for all items and for both groups of candidates. Internal consistency (Cronbach’s α) was calculated for both groups of candidates separately as well as with and without AI-augmented questions to assess their impact on overall reliability. Comparisons across item sources and candidate groups were performed using analysis of variance (ANOVA) or nonparametric equivalents with post hoc testing, where appropriate. To examine differences in item discriminatory power across sources and candidate groups, a two-factor mixed-model ANOVA (split-plot design) was conducted. Item source (previously used bank items, new expert-authored items, new AI-augmented items) was defined as the between-item factor, while candidate group (basic versus specialist candidates) served as the within-item factor. The dependent variable was the discrimination index of each item, operationalized as the corrected item-total correlation. Prior to analysis, assumptions of normality and homogeneity of variances were assessed. In the presence of significant main or interaction effects, post-hoc comparisons with Bonferroni adjustment were applied.

### Analysis of functioning distractors

Distractor functioning was assessed by analyzing the response distribution across all answer options in the 78 single-best answer (A-type) questions (11 new AI-augmented items, 11 new expert-authored items, 56 previously used bank items). Multiple true-false questions (K-prime) were excluded from this analysis. Single-best answer questions included either four or five answer options. All AI-augmented items were constructed with five answer options. Among newly expert-authored items, six included four answer options and five included five answer options. In the previously used bank items, two items were constructed with four answer options, while the remaining items included five options.

In contrast to previous studies reporting non-functioning distractors (NFDs) as an indicator of distractor quality [10, 11], we focused on functioning distractors (FDs). Functioning distractors were defined as incorrect answer options selected by at least 5% of candidates [19]. This approach was chosen because the item pool included both four- and five-answer-option items, resulting in a varying number of distractors per item. As the calculation and comparison of absolute numbers of NFDs assumes a constant number of distractors, reporting FDs provided a more comparable and methodologically appropriate metric across item formats.

For each item, functioning distractors (FDs) were identified and counted. The number of FDs per item was recorded to compare the functional quality of distractors across item sources (previously used bank items, new expert-authored items, and new AI- augmented items). Two and more FD were grouped into one category to avoid cells with no observations.

## Results

### Difficulty across item sources and candidate groups

**Fig. 2:**
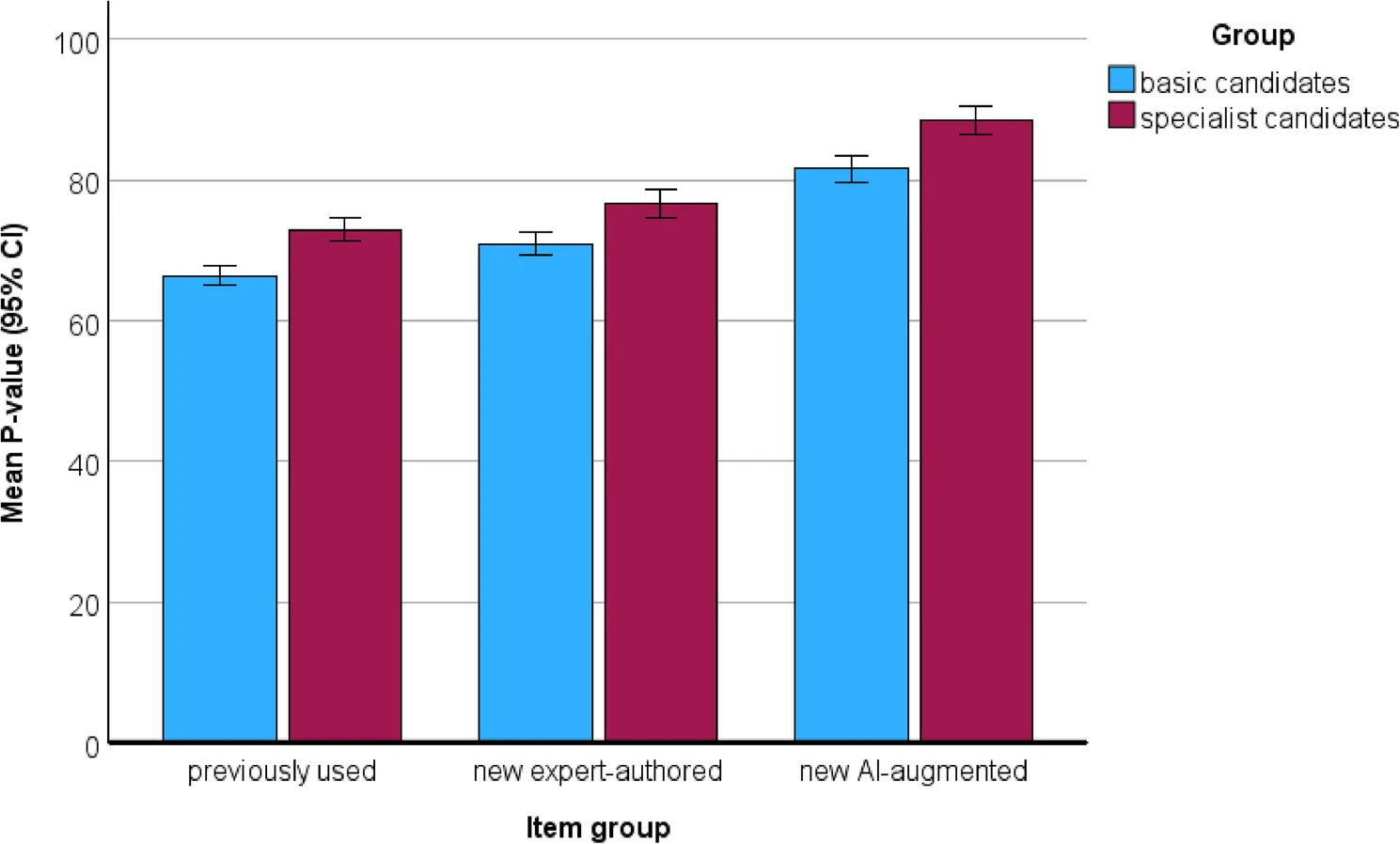
Estimated mean P-values across item groups (previously used bank items, new expert-authored items, new AI-augmented items) and candidate groups (basic candidates and specialist candidates)

Mean P-values, expressed as percentage correct answers, differed significantly across item sources $\:\left({F}_{\left(2,\:604\right)}=297.187,\:p<0.001,\:{\eta\:}_{p}^{2}=0.496\right)$ and candidate groups $\:\left({F}_{\left(1,\:302\right)}=37.737,\:p<0.001,\:{\eta\:}_{p}^{2}=0.111\right)$. P-values pooled across both candidate groups were highest for new AI-augmented items (84.7%, SD=12.7%), followed by newly expert-authored items (73.5%, SD=11.7%) and previously used bank items (69.4%, SD=10.0%). Post-hoc analysis with Bonferroni correction confirmed significant differences in mean P-values across all three item categories. Mean P-values also differed between candidate groups. Items showed significantly higher mean P-values for the specialist candidates’ group (*n*=138, *p*=75.0%, SD=7.5%) than for the basic candidates’ group (*n*=166, 68.5%, SD=10.0%) across all item groups (Fig. 2). No interaction effect between item group and candidate group was observed $\:\left({F}_{\left(2,\:604\right)}=0.377,\:p<0.665,\:{\eta\:}_{p}^{2}=0.001\right)$.

### Discrimination indices

A two-factor mixed-model ANOVA (split-plot design) was conducted to analyze discrimination indices, with item group (previously used bank items, new expert-authored items, new AI-augmented items) and candidate group (basic versus specialist). The analysis showed no significant main effect of item group on discrimination $\:\left({F}_{\left(2,\:112\right)}=0.419,\:p=0.659,\:{\eta\:}_{p}^{2}=0.007\right)$. Compared with expert-authored items (bank items and new items), AI-augmented items demonstrated a comparable ability to distinguish between higher- and lower-performing candidates. However, there was a significant main effect of candidate group $\:\left({F}_{\left(1,\:112\right)}=11.341,\:p=0.001,\:{\eta\:}_{p}^{2}=0.092\right)$, with items administered to basic candidates exhibiting generally higher discrimination indices across all item types compared to items administered to specialist candidates (Table 1). No interaction between item source and candidate group was observed $\:\left({F}_{\left(2,\:112\right)}=0.396,\:p=0.674,\:{\eta\:}_{p}^{2}=0.007\right)$.

**Table 1:** Mean discrimination indices (Mean) and standard deviation (SD) across item groups and candidate groups

Item group		Basic candidates		Specialist candidates	
	*N*	Mean	SD	Mean	SD
Previously used	89	0.23	0.13	0.17	0.13
New expert-authored	14	0.19	0.11	0.15	0.13
New AI-augmented	12	0.24	0.14	0.15	0.16
Total	115	0.22	0.13	0.16	0.13

### Impact on overall test reliability

Cronbach’s α for the complete examination was α = 0.872 for basic group and 0.798 for specialist group, indicating high internal consistency. When AI-augmented items were removed, reliability changed only minimally when test length was considered equal using the Spearman-Brown correction (Δα = 0.002 for basic group and Δα = 0.001 for specialist group) [20, 21].

### Distractor analysis for type-A items

An exact test according to Fisher-Freeman-Halton showed an interrelation between item group and the number of FDs per item in an analysis of a contingency table (*p* = 0.008) for the specialist group (Table 2) [22]. Bonferroni adjusted post-hoc comparisons of cells revealed that AI-augmented questions showed significantly more questions with no FD than expected (std. residual = 3.1), representing the only significant overrepresentation in the table. For the basic group the overall test also reached significance (*p* = 0.012), and the pattern of the observations is analogous to the pattern observed for the specialist group including the overrepresentation in the cell “0 FDs” and AI-augmented questions (std. residual = 3.0).

**Table 2:** Contingency table of item group and number of FDs. Observed and expected frequencies, percentage within item group and standard residuals, displayed for the specialists group

			Item group			
	*N*		previously used	new expert-authored	new AI-augmented	Total
FDs	0	observed (expected)	5 (9.3)	2 (1.8)	**6** **(1.8)**	13
		% of item-group	8.9%	18.2%	**54.5%**	16.7%
		std. residual	-1.4	0.1	**3.1**	
	1	observed (expected)	22 (22.3)	6 (4.4)	3 (4.4)	31
		% of item-group	39.3%	54.5%	27.3%	39.7%
		std. residual	-0.1	0.8	-0.7	
	≥ 2	observed (expected)	29 (24.4)	3 (4.8)	2 (4.8)	34
		% of item-group	51.8%	27.3%	18.2%	43.6%
		std. residual	0.9	-0.8	-1.3	
Total		observed (expected)	56 (56)	11 (11)	11 (11)	78
		% of item-group	100.0%	100.0%	100.0%	100.0%

*FD* Functioning Distractor. Boldface values indicate cells with notable deviations from expected frequencies

## Discussion

In this national high-stakes specialty examination, AI-augmented items were, on average, easier to solve than both new expert-authored and previously used bank items. Discrimination indices were comparable across item sources, and overall reliability showed only minimal change after inclusion of AI-augmented items. At the same time, distractor analysis indicated weaker distractor functioning in AI-augmented items, and a substantial proportion of initially generated AI-augmented items had to be excluded during preliminary screening.

The tendency toward higher P-values (indicating easier items) among AI-augmented items is in line with earlier findings from formative postgraduate examination settings [6, 14]. In contrast to those studies, however, the present analysis was conducted within a real high-stakes context with established review and approval procedures. The results suggest that even when prompts are iteratively refined and expert revision is applied, AI-generated drafts did not consistently align with the optimal difficulty range required for specialty-level assessments. A plausible explanation is that LLMs tend to choose well-known topics and generate well-established patterns with limited narrative depth, which lowers cognitive load and makes the correct answer more apparent [6, 23]. In addition, the multi-stage review process may have contributed to the observed findings, as AI-generated items perceived as overly complex or ambiguous may have been preferentially excluded during review. Consequently, the higher P-values observed among the retained AI-augmented items may partly reflect the effects of item selection in addition to characteristics of AI-assisted item generation itself. At the same time, they reflect the intended use of AI-generated items within a secure, real-world examination setting that includes rigorous expert review and quality assurance procedures.

AI-augmented items were significantly more likely to contain no functioning distractor. When most distractors are rarely selected, the competitive structure of response options is reduced. Prior research suggests that items with a higher number of functioning distractors tend to be more difficult and demonstrate stronger discriminatory properties [19]. A limited number of functioning distractors may therefore partly explain the higher proportion of correct responses observed in AI-augmented items. Differences in the number of answer options should be considered when interpreting distractor performance. However, AI-augmented items showed a disproportionate number of cases with only one functioning distractor despite uniformly including five answer options.

At the same time, distractor functioning does not follow a simple linear relationship with item quality. Items with multiple functioning distractors do not automatically exhibit superior discrimination indices [26]. These findings underscore the importance of careful post-exam analysis in high-stakes specialty examinations to ensure that distractor structure meaningfully contributes to candidate differentiation.

Discrimination indices did not differ significantly between item sources, consistent with previous studies [6, 8, 10, 14]. This suggests that AI-augmented items remained capable of distinguishing between higher- and lower-performing candidates. Across all item types, discrimination was lower in the specialist cohort than in the basic examination group. This pattern aligns with classical test theory: in more homogeneous, high-performing cohorts, restricted score variance limits the maximum attainable discrimination [20, 21]. The clustering of AI-augmented items at higher P-values may therefore be particularly consequential in specialist cohorts. When items are relatively easy for a high-ability group, reduced score spread further attenuates discrimination [24].

Given the observed tendency toward higher P-values in AI-augmented items, preliminary difficulty estimation becomes particularly important during item approval to prevent an unintended reduction in overall examination difficulty. Prior research indicates that estimating item difficulty remains challenging even for experienced reviewers [25]. Automated difficulty prediction tools, as recently explored by Pereira et al., may offer complementary support, particularly if embedded within structured validation workflows rather than applied in isolation [26].

Although the inclusion of AI-augmented items did not substantially affect overall test reliability at the examination level, the high exclusion rate observed during initial screening highlights a different practical concern. A considerable proportion of generated items did not meet predefined standards regarding accuracy, authenticity, or structural quality. Thus, while AI-augmented items can be integrated without compromising aggregate psychometric indices, substantial revision effort remains necessary before their use in high-stakes examinations.

An additional observation from the development process was that broadly framed prompts requesting multiple items within a general subject area did not result in a sufficiently broad or proportionally distributed coverage of all relevant topics. Instead, the model tended to generate items clustered around prototypical or frequently represented themes, limiting thematic diversity. Achieving acceptable domain coverage therefore required substantial iterative refinement and explicit specification of underrepresented areas. Only through targeted re-prompting and expert-guided adjustment could alignment with predefined examination standards be achieved. This observation is consistent with prior reports emphasizing that prompt design and iterative refinement substantially influence the quality of AI-generated educational content and that expert input remains essential in assessment development [7, 12, 13, 15]. In its current form, large-scale autonomous generation of thematically balanced item sets without structured domain guidance does not appear feasible.

Further work should examine whether targeted distractor refinement, alternative prompting strategies, or hybrid AI–expert workflows can improve calibration and reduce exclusion rates. Systematic documentation of revision processes may also help to identify recurring patterns in AI-generated drafts and inform more efficient quality assurance strategies.

## Limitations

This study has several limitations. It was conducted within a single specialty and national high-stakes examination context, which limits the generalizability of the findings to other disciplines or assessment formats. The number of AI-augmented items included in the examination was relatively small, reflecting the first implementation of AI-generated items in a high-stakes examination. Among the review-approved AI-generated questions, preference was given to items judged to be of the highest educational and clinical quality while ensuring appropriate blueprint coverage and avoiding content overlap. Consequently, the included items may not fully represent all review-approved AI-generated questions. Future research should examine larger and more diverse samples of AI-generated items across different specialties and examination settings.

AI-generated items were produced using a single model (ChatGPT-4o) via the browser-based interface. Model parameters could not be explicitly controlled or archived, limiting technical reproducibility. Although necessary to safeguard examination integrity, the multi-stage review process did not allow systematic documentation of all revisions. Consequently, the specific contribution of revisions to item difficulty and distractor performance could not be quantified.

## Conclusion

Our findings suggest that AI-augmented multiple-choice questions can be incorporated into high-stakes specialty examinations when embedded within rigorous expert review and quality assurance processes. However, AI-augmented items did not consistently achieve the target difficulty range for specialist-level assessment and were more likely to exhibit limited distractor functioning. Although overall reliability remained stable, the substantial exclusion rate during initial screening underscores the continued necessity of human oversight.

AI may serve as a drafting tool in assessment development; however, integration into established review procedures, careful calibration, and systematic post-exam analysis remain essential to ensure psychometric robustness in high-stakes contexts.

## Data Availability

The datasets generated and analyzed during the current study are not publicly available due to examination security restrictions but may be available from the corresponding author on reasonable request and subject to approval by the responsible examination authorities.

## Abbreviations

AI: Artificial Intelligence
LLM: Large Language Model
MCQ: Multiple-Choice Question
FD: Functioning Distractor
NFD: Non-Functioning Distractor
OB/GYN: Obstetrics and Gynecology
